# Nurses’ Competencies in Disaster Nursing: Implications for Curriculum Development and Public Health

**DOI:** 10.3390/ijerph110303289

**Published:** 2014-03-20

**Authors:** Alice Yuen Loke, Olivia Wai Man Fung

**Affiliations:** 1 School of Nursing, The Hong Kong Polytechnic University, Hung Hom, Kowloon, Hong Kong; 2 Nethersole School of Nursing, The Chinese University of Hong Kong, New Territories, Hong Kong; E-Mail: Oliviafung@cuhk.hk

**Keywords:** competencies, disaster nursing, disaster nursing curriculum

## Abstract

The purpose of this study was to explore Hong Kong nurses’ perceptions of competencies required for disaster nursing. Focus group interviews and written inquiry were adopted to solicit nurses’ perceived required competencies for disaster care. A total of 15 nurses were interviewed and 30 nurses completed the written inquiry on their perceived competencies related to disaster nursing. The International Council for Nurses’ (ICN) framework of disaster nursing competencies, consisting of four themes and ten domains, was used to tabulate the perceived competencies for disaster nursing reported by nurses. The most mentioned required competencies were related to disaster response; with the ethical and legal competencies for disaster nursing were mostly neglected by nurses in Hong Kong. With the complexity nature of disasters, special competencies are required if nurses are to deal with adverse happenings in their serving community. Nurses’ perceived disaster nursing competencies reported by nurses were grossly inadequate, demonstrating the needs to develop a comprehensive curriculum for public health. The establishment of a set of tailor-made disaster nursing core competencies for the community they served is the first step in preparing nurses to deal with disastrous situations for the health of the public.

## 1. Introduction

With disasters occurring more frequently threatening people around the world, the need to prepare nurses for disaster has never been greater [1]. Nurses should be equipped with the necessary knowledge and abilities to work in a disaster and to meet the needs of the respective serving community. However, more than 80% of nurses who volunteered to serve for a disaster event had no previous experience in disaster response [2]. It was recommended by the World Health Organization (WHO) that all nations, no matter how frequent (or infrequent) the happenings, should prepare healthcare workers for a disaster. Nevertheless, most nurses were inadequately prepared for disaster [3]. It is only through education and training can nurses can be equipped with the competencies required during disasters. 

As the largest manpower group in the healthcare team, nurses play an important role in disaster care. Nurses’ roles are not only in the emergency phase of a disaster, to rescue life and safeguard the health of the disaster sufferers, but in fact, nurses have special roles in disaster preparedness and aftermath long-term recovery [4]. By providing leadership and guidance in different phases of a disaster, nurses can safeguard the health of the general public and reduce death tolls [5]. 

Nurses need to be competent in order to deal with disastrous situations. Competency refers to the actual performance of a person in a specific role, in a given situation [6]. It is defined as a combination of the knowledge, skills, abilities and behavior needed to carry out a job or special task [7]. Although there are many sets of competencies being developed to prepare healthcare workers to respond to disaster, they have been found inconsistent and imprecise. Disaster nursing core competencies specific to general nurses were limited or not verified. Further effort has to be made and directed toward the development of an accepted and adapted framework of competency for universal disaster nursing education [4].

The awareness of disaster preparedness and competencies among Hong Kong nurses is generally weak [8,9]. With limited research, training and education in the field of disaster nursing in Hong Kong, it is important to understand nurses’ perceived competencies in disaster care prior to curriculum development. A better understanding of nurses’ perceived competencies and their learning needs for disaster nursing is the first and essential step if nurse educators and health care administrators are to launch a disaster nursing course/continuing education program that meet the needs of nurses. This study adopted the framework of disaster nursing and key competencies delineated by International Council for Nurses and World Health Organization [10] as the standards to scrutiny Hong Kong nurses’ level of competencies for disaster nursing. 

The ICN Framework of Disaster Nursing Competencies 

With an urge to develop a set of core competencies for disaster nursing education, the ICN launched a framework of disaster nursing competencies in 2009 for general nurses. It aims to work as a common set of competencies in disaster nursing for the global nursing workforce and to provide clarification of nurses’ role in disasters [10]. The framework of nursing competencies should be applicable globally and the content can be modified to be culturally specific for different regions and places. It is also emphasized that in-country interpretation of the framework and regular review of the competencies is important to ensure relevancy to the community served. 

The ‘‘ICN framework of disaster nursing competencies’ was built on the ‘‘ICN framework of competencies for general nurse’, which has been widely used as a guideline of nurses’ competencies at an international level. The ICN disaster nursing competencies framework was developed according to the competencies in the areas of public health, mental health, healthcare workers, emergency managers, nursing and disaster nursing. The framework also based on the two sets of widely-used disaster competencies for general nurses, the Nursing Emergency Preparedness Education Coalition [11] for mass casualty incidents and the Centre of Excellence, University of Hyogo (Kobe, Japan) [12] Disaster Competencies [10]. 

The ICN framework of disaster nursing competencies consists of four areas in the continuum of disaster management that corresponds to the four stages of disaster: the prevention, preparedness, response, and recovery stages. The four areas include ten domains, consists of a total of 130 items, in which nurses play numerous and multiple vital roles in disaster care and management [10]. 

## 2. Study Methods

The aim of this study was to identify nurses’ perceived competencies for disaster. The study was divided into two parts: focus group interviews and a written inquiry. The focus group interviews were to explore the competencies and knowledge of disaster nursing readily reported by the nurses with minimal guided questions or information. They were, given an explanation of what entails a disaster at the beginning of the interview sessions. The written inquiry was then followed, which consisted of a table specifying the four stages of disaster according to the ICN disaster framework as prompt, and nurses were asked to write down what they considered were the nursing competencies required for each of the four stages of disaster. 

### 2.1. Participants/Sample Selection

The study focuses on nurses working in the medical/surgical, critical care and community health settings. These three groups of nurses were selected for their representativeness of the major nursing workforce during disaster. Medical and surgical nurses constitute the largest group in the nursing workforce; their competencies represent the competencies of general nursing for disaster care. The critical care nurses work in high-dependency units including the intensive care unit and the emergency department; they are the first to response and help victims in disasters. The community health nurses work closely with the general public, and are most likely to understand the needs of the community they served. The perceived disaster nursing competencies of these three groups of nurses could provide a better understanding of the overall competencies level of Hong Kong nurses. 

Nurse leaders from specialty associations, with extensive experience in their respective clinical specialty, were recruited for group interviews. There were four to six nurse leaders from the three specialties in each of the interview groups. Convenience sampling was used for the written inquiry. The participants were referred by senior nursing staff in hospitals or clinical settings. A total of thirty participants, ten from each specialty, were invited to complete the written open-ended inquiry.

### 2.2. Group Interviews

Group interviews were conducted separately among nurses in the three specialities. They were asked to state the competencies they considered to be necessary to handle disasters in their specialty area. Simple explanation on the definition of disaster was given. The interviews were tape-recorded and transcribed within one week after the interview. The interviewer repeated and summarized the participants’ stated competencies to get an immediate indication of their agreement that the summary was a true and comprehensive list of their responses [13]. To ensure accuracy, these initial sets of compiled competencies were checked by two nurses, who verified the tabulated competencies separately to ensure exactness [14]. A table is used to summarize the nurses’ perceived disaster competencies, which were tabulated according to the specialty areas and according to the domains of the ICN framework of disaster nursing competencies. 

### 2.3. Written Inquiry

Written inquiry was conducted to further substantiate the findings of the group interviews. Written forms of data collection have the advantage of putting the participants under less pressure and giving them time to ponder upon what are being asked. A table listing the four main disaster stages and ten domains of disaster nursing competencies according to the ICN disaster nursing competencies framework [10] was given to the nurses in the three specialty groups (general medical/surgical, critical care and community health) to further substantiate information collected from the group interviews. The questionnaire was distributed and collected by the researchers in person.

### 2.4. Data Analysis and Establishing Trustworthiness

Tabulation of the group interview responses was done in a systematic, sequential, verifiable way separately by the two researchers for the confirmability of the data. The interviews were audio-recorded and also preserved for repeat auditing. The transcription and tabulation of the data was done within one week of the interviews. The content (stated competencies) was categorized according to the four areas and ten domains of the ICN framework of disaster nursing. The tabulation were discussed and verified by the two researchers for rigor of the study [14].

For the written inquiry, the written responses of the specific competencies were directly categorized and tabulated under the four stages and 10 domains of the ICN framework of disaster nursing. The findings of the three interview groups and three written inquiry groups were merged in one table for ease of comparison.

### 2.5. Ethical Considerations

Ethical approval was obtained from the institute where the researchers work, and the conduct of the study was according to the stipulated regulation of the institute. The nurse participants were given an explanation of the aims of this study, and assured that participation was voluntary. All studies were completed in anonymity so that their personal identity could not be identified. Participants who willing to join the group interviews and being recorded, or complete the written inquiry were considered giving an implied consent to the study. The audio-recordings, notes and interview transcripts were anonymous, and contained only information on the nurses’ specialties and work experience.

## 3. Results

### 3.1. The Participants

A total of 45 nurses participated in the study, the majority of them were female (82%). Group interviews of 4–6 nurse leaders from the three specialties were conducted separately to explore nurses’ perceived competencies in disaster management. All were experienced nurses with 3 to 23 years of experience, averaging of 17.2, 13.8, and 15.3 years of experience in the medical/surgical, critical and community specialty respectively (Table 1).

A total of 30 participants, 10 from each specialty, were invited in the written inquiry study. They had 3 to 28 years of working experience in their specialty, with an average of 16.4, 14.7, and 10.7 years of experience in the medical/surgical, critical, and community specialty.

**Table 1:** Clinical experience of the participants.

Method of Data Collection	Medical/Surgical	Critical Care	Community Unit
Focus groups			
No. of participants	6	5	4
Year of Experiences	9–23 years	3–20 years	8–23 years
Means	17.2 years	13.8 years	15.3
Open ended inquiry			
No. of participants	10	10	10
Year of Experiences	3–24 years	4–28 years	3–15 years
Means	16.4 years	14.7 years	10.7 years

Hong Kong Nurses’ Perceived Competencies for Disaster

Hong Kong nurses’ perceived disaster competencies solicited from group interviews and written inquiry were tabulated under the four main areas (stages of disaster) of the ICN framework of disaster nursing competencies. The findings are discussed below according to the four stages of disasters (Table 2).

### 3.2. Prevention and Mitigation Competencies

From the group interviews, there was consensus of nurses from all three specialties considered: ‘‘risk assessment and management”, ‘‘provision of appropriate protective materials”, ‘‘development and planning of organizational guidelines or protocol for disaster management”, and ‘‘planning for specific incident management” as required disaster nursing competencies. However, only the critical care and community health nurses regarded ‘‘adhering to infection control principles” and ‘‘the need for contingency plans for disasters as important competencies in disaster prevention. 

**Table 2:** Hong Kong nurses’ perceived competencies for disaster care reported in focus group interviews (*n* = 15) and written inquiry (*n* = 30).

The 4 areas and 10 domains of the ICN Framework of Disaster Nursing Competencies		Focus group interviews		Written inquiry			
Prevention/Mitigation Competencies (total = 23)	Competencies	Medical surgical nurses (*n* = 6)	Critical care nurses(*n* = 5)	Community nurses(*n* = 4)	Medical surgical nurses(*n* = 10)	Critical care nurses(*n* = 10)	Community nurses(*n* = 10)
1.Risk Reduction, Disease Prevention and Health PromotionRisk Reduction and Disease Prevention (7)Health Promotion (5)	-Risk assessment and management	√	√	√	√	√	√
	-Adhere to infection control principles		√	√	√	√	√
	-Provide appropriate protective materials	√	√	√			√
	-Knowledge in disaster/illness and primary health care			√		√	√
	-Practice personal hygiene				√	√	
	-Preparation of health staff and the public in preventing disaster				√		
	-Provide relevant reference materials					√	
	-Work in a multidisciplinary approach to care	√					
	-Understand public health/epidemiology/vaccination						√
	-Understand causes/mechanisms/prevention of disaster				√		
2. Policy Development and Planning (11)	-Development of organizational and unit guidelines/protocol	√	√	√	√	√	√
	-Provide contingency planning		√	√	√	√	√
	-Plan (with protocol) for specific incident management	√	√	√			
	-Infection control policy				√	√	√
	-Public health policy				√		√
	-Regular review of protocol						√
	-Risk management policy					√	
	-Fire safety and evacuation plan				√		
	-Quality and safety guidelines					√	
	-Occupational health and safety					√	
	-Manpower deployment plan		√		√		
3. Ethical Practice, Legal Practice and Accountability3.1. EthicalPractice (7)3.2. LegalPractice (5)3.3. Accountability (5)	-Professional obligation to include disaster prevention, response, plan and recovery in practice				√		
	-Follow code of conduct				√		
	-Legal liability and government overall disaster planning				√		
	-Establish, understand and reinforce laws on disaster prevention					√	
	-No discrimination based on gender, religion, nationality, social status					√	
	-Compliance with Privacy Ordinance					√	
	-Human dignity is important					√	
	-Patient charter					√	
	-According to HK Nursing Council code of conduct						√
	-Provide complaint system						√
	-Practice according to professional standard						√
	-Knowledge of legal practice						√
4.Communicationand Information Sharing (12)	-Develop communication skills	√	√	√	√		
	-Debriefing/incident reporting and meeting	√	√	√	√		
	-Use of various tools for communication		√				
	-Establish fast and accurate communication of information system among government and non-government organizations, the community, hospitals and wards				√	√	√
	-Press release of information				√	√	
	-Yearly review, share information with other countries					√	
	-Use various tools for communication				√	√	√
	-Familiarity with the data disclosure, communication, and information according to the guidelines						√
5. Education and Preparedness (12)	-Provide: drill/audit/talk	√	√	√	√		
	-Knowledge and skill in different disaster situations	√	√	√			
	-Leadership skills	√	√	√			
	-Understand role in disaster assignment	√		√			
	-Basic life support, CPR skills		√				
	-Updating information about new diseases				√		
	-Training in IT and communication skills				√		
	-Skills in psychological intervention				√		
	-Understanding of the nature of disasters				√		
6. Care of the Community (11)	-Allocation/distribution of? limited resources	√	√	√		√	√
	-Knowledge of prioritizing care	√		√			
	-Active participation in rescuing activities				√		
	-Collaboration in community resources/voluntary service					√	
	-Reminder cards for management of specific disasters		√				
	-Care for the safety, security, access of food and water, medical care, temporary shelters, *etc.*					√	
	-Provide talks and a hotline in service area						√
	-Community services for different groups, e.g., geriatric assessment service						√
7. Care of Individuals and Families7.1. Assessment (7)7.2. Implementation (18)	-Personal safety, escape route-Disaster preparedness plan for self and family			√			
	-Establish logistics for the care of victims				√		
	-Perform holistic care				√		
	-Help desk for enquiries				√		
	-Form critical incident support team					√	
	-Perform holistic care					√	
	-Familiar with different available resources, support network and referral for victims and families						√
	-Liaison with related social support					√	
	-Multidisciplinary approach to care					√	√
8. Psychological Care (9)	-Psychological first aid and crisis intervention	√	√	√		√	
	-Psychological assessment and counseling therapies for stressed staff and victims (form sharing groups)	√			√	√	√
	-Knowledge and skills in psychological/social aspect					√	
	-Adopt a multidisciplinary approach to care					√	√
	-Introduce coping skills and knowledge on disaster care						√
	-Post-Traumatic Stress Disorder care						√
9. Care of Vulnerable Populations (6)	-Care of neglected groups with special needs			√	√		
	-Work in a multidisciplinary approach to care (voluntary service referral)					√	√
	-Understanding the needs of vulnerable populations					√	
	-Special care and education for populations particularly vulnerable to disasters, *i.e.*, those with chronic illness, pregnant women and the fragile elderly, people with sensory disabilities				√	√	
	-Ability to identify vulnerable populations						√
10. Long-Term Individual, Family and Community Recovery10.1. Individual and Family Recovery (7)10.2. CommunityRecovery (8)	-Evaluation and planning in prevention and management during and after a disaster	√					√
	-Restoration of normal service				√		
	-Work and support by resources in a multidisciplinary approach to care					√	
	-Knowledge and skill in psychological and long-term care					√	
	-Participate in the development of an ordinance and community-wide policies to speed up recovery from disaster				√		
	-Collaboration between community and family for post-disaster recovery						√
	-Learn and share					√	
	-Systematic long-term care for disaster recovery						√
	-District support and resource allocation						√

From the written inquiry, nurses of the three specialties identified that the ‘‘risk assessment and management” and ‘‘adherent to infection control principles” as important in disaster prevention. Only the community healthcare nurses considered ‘‘vaccination and epidemiology” as required competencies in disaster prevention. Whereas, the medical/surgical nurses expressed their concern on the ‘‘plan of manpower deployment”, the critical care nurses concern about ‘‘occupational health and safety”, and the community nurses highlighted the significant of ‘‘public health policy in disaster prevention planning”.

### 3.3. Preparedness Competencies

In the interviews, “the knowledge of legal and ethical aspects of disaster care” was not addressed by any of the nurses. While all nurses across the three specialties were well aware of the importance of “developing good communication skills” and that “debriefing and incident reporting” was necessary. Education and preparedness for disaster related to “drills, audits or talks”, and the “knowledge and skills in different disaster situation” were reported by nurses in all three specialties as required competencies. 

The findings from written inquiry were somewhat different. All nurses recognized that ‘‘professional obligation” and ‘‘code of conduct” are the required competencies in disaster care. The medical/surgical nurses considered ‘‘legal liability” and ‘‘professional code of conducts” should be reinforced in disaster care. Critical care nurses acknowledged ‘‘human dignity” and ‘‘absence of discrimination” in disaster care, while the community nurses opined that there is need for ‘‘a complaint system” and knowledge of ‘‘legal practice”. Nurses from all three specialties considered the need for ‘‘communication skills and reporting systems” and recommended the ‘‘use of various tools for communication and information sharing”. Only the medical/surgical nurses were able to point out the importance of ‘‘updating information about new diseases”, ‘‘training in information technology and communication skills”, ‘‘skills in psychological intervention”, and ‘‘understanding the nature of disaster” as essential competencies. 

### 3.4. Response Competencies

The interviews revealed that nurses from all three specialties regarded that ‘‘allocation and distribution of resources” during a disaster needed to be addressed, and they were concerned about ‘‘psychological care and crisis intervention” in a disaster. Both medical/surgical and community nurses regarded it necessary to have ‘‘knowledge of prioritizing care”. Critical care nurses suggested the use of ‘‘reminder cards for management of specific disasters”, in order to enable efficient triage and prioritize care. Only the community nurses aware of the fact that the establishment of ‘‘escape routes and personal safety” as disaster preparedness for the community. They also acknowledged that there is a need to care of ‘‘vulnerable groups as a neglected population with special needs”. 

From the written inquiry, the medical/surgical nurses addressed the need to establish ‘‘logistic for care of victims”, ‘‘perform holistic care”, and ‘‘help desk for enquires”. The critical care and community nurses stressed the need for ‘‘multidisciplinary approach to care”. Community health nurses emphasis the need for psychological care, including ‘‘introduce coping skills and knowledge on disaster care”, and ‘‘post-traumatic stress disorder care”. 

### 3.5. Recovery and Rehabilitation Competencies

The findings of the interviews showed that only medical/surgical nurses talked about the important to have a plan for ‘‘evaluation and planning in management during and after a disaster”. The findings of the written inquiry revealed that nurses in all three groups addressed the competencies of post-disaster care. Medical/surgical nurses identified the need to learn ‘‘restoration of normal services. Critical care nurses noted the significance of ‘multidisciplinary approach’ in the recovery stages of a disaster and the ‘knowledge of psychological care for victims’. The community nurses emphasis the ‘collaboration between community and family for post-disaster recovery’, systematic long-term care for disaster recovery”, and ‘‘district support in resources allocation”, as well as ‘‘evaluating and planning for future disaster management”. 

## 4. Discussion

There is a global need for all healthcare workers to be prepared and be competent in disaster care. Previous studies have found that most nurses were not adequately prepared [8,9]. The findings of the study indicated that nurses were not aware of their roles in preparing the community or the vulnerable population for disaster. In order to be prepared and be competent for disaster, all nurses should be equipped with knowledge and skills for disaster care through continue education and training. ‘‘Disaster nursing” has not yet been established as a core topic/subject to be included in nursing programs in Hong Kong, though it is a global demand for the inclusion of this component of disaster care in our education program. The findings of this study provide a clearer picture of the inadequate preparations of nurses for disaster, in that it provides nurse educators and/or health care administrators a guide to delineate a tailor-made education program for nurses. 

The results of this study showed that Hong Kong nurses have some understanding of the needed competencies in ‘‘prevention, preparation, response, and recovery” phases of disaster care. In fact, the ICN has suggested in its disaster nursing framework that more attention is needed related to planning and preparation, as well as the understanding of the whole disaster management process. It is reflected that although there were quite a number of studies have focused on disaster response, there are also some studies conducted in Hong Kong on the disaster preparedness of families with young children [15] and elderly people [16] in Hong Kong. For post-disaster care, a study was also conducted that explore the experience of China nurses after the Sichuan earthquake rescue [17]. 

In the stage of ‘‘prevention and mitigation phase” of disaster care, the most neglected competencies were the ‘‘preparation of health staff and the public in preventing disaster”, ‘‘regular review of protocol”, and ‘‘quality and safety guideline”. In the ‘‘preparedness” phase, the less mentioned competencies were ‘‘practice according to professional standard”, ‘‘updating information about new diseases”, ‘‘training in information technology and communication skill”, ‘‘skills in psychological intervention”, and ‘‘understanding of the nature of disaster”. During the response phase, the competencies related to ‘‘forming critical incident support team”, familiar with different resources, support network, and referral for victims and families’, and the ‘‘ability to identify vulnerable populations”, ‘‘post-traumatic stress care”, and ‘‘care of special population with special needs”, were not attended to. In the recovery phase, the competencies of ‘‘evaluation and planning of management after a disaster”, ‘‘restoration of normal service”, ‘‘collaboration between community and family for post-disaster recovery”, ‘‘systematic long-term care for disaster recovery” require more attention. If health workers are to prepare for disasters, all these competencies needed to be included in the all nursing curriculum and continuing nursing program.

None of the nurses in this study mentioned their own preparation and that of their families for disaster. A study in Hong Kong have found that the nurses’ preparedness of their families affect their willingness to report to work during disaster [8]. Nurses, as well as all other health professionals, should be aware of the importance of being prepared individually and in their families, so that they can be ready to provide care during disaster, and to protect life. The message of being prepared for disaster should be conveyed to all members in the community through public education. 

The special needs of vulnerable groups should be attended to in disaster to reduce damaging effects on health of the population and the death toll. Nurses should understand the risks and the needs of these special populations in their serving community and equip themselves to support them in disaster. Nurses should identify the vulnerable populations in the community, assist them in their special needs with special items available at home, the basic survival skills, where and how to get help. Checklists and education talks should also be developed and offered targeted these vulnerable groups.

## 5. Conclusions

This study explored the perceived disaster competencies of Hong Kong nurses and provides the needed background to inform educational needs. The findings of the study also provide hospital administrators the need to develop continuing education to prepare their nurses with the competencies for disaster care in their respective specialties. The study also shows that a context appropriate set of disaster nursing competencies is needed for Hong Kong nurses. The ICN framework can be used as a guide; with further modification and refinement to increase the applicability and validity of the competencies for the community we served [10,18]. 

With the increase frequency of disaster happenings globally, the need for education and training preparation is to be emphasized. A set of core competencies has also been defined as a starting point for delineating expected competency of health professionals in disaster medicine and public health [19]. Nurses should be well adequately prepared with knowledge and skills for management of disasters, starting early from their basic training and reinforcement in their on-the-job continuing training. Nurses, in all specialties, should be equipped for all competencies for disaster prevention, preparedness, response, and recovery phases. The public should also be prepared through disaster awareness promotion activities and health talks. The development of a comprehensive disaster nursing curriculum can protect our people in the community we served and shouldering the international responsibilities during disaster events in our nearby countries. In fact, disaster simulation has been used as an educational strategy to prepare nursing students for disaster respond, and has been incorporated into the undergraduate nursing curriculum [20], The simulation was found to increase nursing students’ understanding of disaster preparedness, increase their ability and confidence to handle disastrous situations and working in teams. 

In this early stage of developing disaster nursing in different societies, exploration of educational need, further research, and establishment of a set of core competencies for disaster nursing appropriate for the societal context are essential.

## 6. Limitations

The disaster nursing competencies standard delineated by ICN, and referenced by all nurses worldwide, was adopted as the framework to evaluate the perceived competencies of nurses in Hong Kong for disaster. The categorization of key competencies reported by nurses align with the ICN framework of disaster nursing competencies may be arbitrary, but was merely a practical way to estimate the nurses’ level of competencies, further research is needed to identify nurses’ competent level in meeting the needs for disaster nursing. This study focused on nurses from three specialties may not be generalizable to other nurses and further study is needed to include other nurses from other specialties.
